# Socioeconomic Disparity in the Prevalence of Objectively Evaluated Diabetes Among Older Japanese Adults: JAGES Cross-Sectional Data in 2010

**DOI:** 10.2188/jea.JE20170206

**Published:** 2019-08-05

**Authors:** Yuiko Nagamine, Naoki Kondo, Kenichi Yokobayashi, Asami Ota, Yasuhiro Miyaguni, Yuri Sasaki, Yukako Tani, Katsunori Kondo

**Affiliations:** 1 Department of Public Health, Graduate School of Medicine, Chiba University, Chiba, Japan; 2 Department of Health Education and Health Sociology, School of Public Health, the University of Tokyo, Tokyo, Japan; 3 Homecare Clinic, Hiroshima, Japan; 4 Division of Health and Nutrition, University of Niigata Prefecture, Niigata, Japan; 5 Department of Gerontological Evaluation, Center for Gerontology and Social Science, National Center for Geriatrics and Gerontology, Aichi, Japan; 6 Department of International Health and Collaboration, National Institute of Public Health, Saitama, Japan; 7 Department of Global Health Promotion, Tokyo Medical and Dental University (TMDU), Tokyo, Japan; 8 Research Fellow of Japan Society for the Promotion of Science, Tokyo, Japan; 9 Center for Well-being and Society, Nihon Fukushi University, Nagoya, Japan

**Keywords:** socioeconomic status, diabetes mellitus, sex differences, elderly adults, Japan

## Abstract

**Background:**

Studies on sex-specific socioeconomic gradients in objectively evaluated diabetes among older adults are scarce.

**Methods:**

We used cross-sectional data of 9,893 adults aged 65 years and older in Aichi Prefecture without long-term care insurance from the Japan Gerontological Evaluation Study (JAGES) in 2010 (Response rate: 66.3%). We collected demographic, socioeconomic (income, years of education, and longest occupation) and behavioral information using a mail-in self-reported survey. Blood samples for the objectively evaluated diabetes and self-reported medical history were collected at annual municipal health checkups. Poisson regression analysis stratified by sex with multiple imputations was conducted to calculate prevalence ratio and 95% confidence interval.

**Results:**

A clear income gradient in diabetes prevalence was observed among women, from 11.7% in the lowest income quartile (Q1) to 7.8% in the highest (Q4). Among men, the findings were 17.6% in Q1 to 15.1% in Q4. The prevalence ratios for diabetes with incomes Q1 to Q4 were 1.43 (95% confidence interval [CI], 1.07–1.90) for women and 1.16 (95% CI, 0.90–1.50) for men after adjusting for age and other socioeconomic factors. Even after adjusting for marital status, body mass index, other metabolic risk factors, and lifestyle factors, the income-based gradient remained among women. Education and occupation were not significantly associated with diabetes in the study population.

**Conclusions:**

Only women showed an income-based gradient in diabetes. Monitoring income gradient in diabetes is important in public health actions, even in older populations. Future longitudinal and intervention studies should evaluate the causal link of income to diabetes onset, determine the mechanisms of the potential sex differences in the income/diabetes association, and identify ways to mitigate the income-based inequality.

## INTRODUCTION

According to global reports on diabetes 2016, about 422 million adults were living with diabetes worldwide in 2014. During the past 4 decades, the global prevalence of adult diabetes has nearly doubled, increasing from 4.7% to 8.5%.^1^ In Japan, the National Health and Nutrition Survey reported that 16.2% of men and 9.2% of women aged over 20 years were suspected to have diabetes in 2015.^1^ About 3.2 million people (1.8 million men and 1.4 million women) received treatment for diabetes in 2014,^2^ and 70% were over 65 years old.

Socioeconomic disparities in diabetes prevalence and incidence have been well documented in Western countries^3^^–^^11^ and some Asian countries, including South Korea,^12^ China,^13^^,^^14^ Taiwan,^15^ and Japan.^16^^,^^17^ Except for one study in China,^14^ inverse relationships between socioeconomic status (SES) and diabetes prevalence or incidence have been observed according to occupational class,^3^^,^^18^^,^^19^ income level,^10^^,^^18^^,^^20^ and educational attainment.^3^^,^^10^^,^^18^^–^^22^ However, few studies have investigated the social gradient in diabetes among older adults, and the findings of studies using data among older populations have been inconsistent with respect to the association between SES and diabetes. For example, a Finnish study has shown that the low-income group had a higher diabetes prevalence compared with the high-income group among women, whereas among men the high-income group was more likely to have diabetes.^6^ Studies in Germany^23^ and the United Kingdom^5^ have shown that neither income, education, nor occupation was associated with the incidence of diabetes.

To date, many studies have suggested sex differences in the association between SES and diabetes. Most studies have reported more obvious social gradients in diabetes among women than men in Western countries.^3^^,^^6^^,^^18^^–^^20^^,^^22^ In Asian countries, only Lee et al in South Korea investigated sex differences in the association between SES and diabetes, similarly showing a stronger SES-diabetes association among women than men.^21^ No evidence of sex differences in the association between SES and diabetes has been reported from other parts of Asia, including Japan.

These studies, other than the study in the United Kingdom (*N* = 7,432), are not large (Finland: *N* = 379, Germany *N* = 1,223), which may be a limitation in detecting the between-group gaps. Moreover, in the recent studies, the definitions of diabetes vary. For example, diabetes was defined using self-reports,^6^ first diabetes medication prescribed,^5^ and oral glucose tolerance tests.^23^ Specifically, self-report of having diabetes could induce reporting bias. The validity study by Goto et al found that positive predictive value of self-report diabetes was 75.7%, whereas negative predictive value was 96.5% in the Japanese population.^24^ The bias may go toward null on the association between SES and diabetes prevalence, given that health-conscious people with high health literacy recognize their health status more accurately. According to a Japanese nationally representative survey, health-conscious people are likely to be more educated.^25^ To our knowledge, no studies have investigated the association between objectively diagnosed diabetes and SES among the older population.

Therefore, the purpose of this study was to investigate (1) whether there is an association between SES and diabetes prevalence among Japanese older adults, and (2) whether there is a sex difference in this association, using large-scale cross-sectional data with objectively measured biomarkers of diabetes.

## METHODS

### Study participants

We used cross-sectional data of the 2010 wave of the Japan Gerontological Evaluation Study (JAGES). In 2010, in JAGES we sent the questionnaires to 169,215 community-dwelling individuals over 65 years without long-term care insurance. From 31 municipalities in 12 out of 47 prefectures throughout Japan, participants were randomly selected from the public residence registries in 15 large municipalities; in the 16 smaller municipalities, all eligible residents got the mail-in survey. In total 112,123 subjects answered the questionnaire (response rate: 66.3%). After excluding the individuals with missing in demographic characteristics, 102,869 subjects were valid for analysis. Since JAGES exclude long-term care insurance takers from the study participants, we cannot compare the characteristics of the participants with the national census directly. However, the sex ratio of the total JAGES 2010 individuals was mostly comparable to that of the national census (national census: 42.6% men, 57.4% women; JAGES2010 data: 45.9% men, 54.1% women). JAGES female population was younger than that of the national census (national census: 32.2% age group ≥80; JAGES2010: 21.5% age group ≥80), whereas the age structure of the male population was mostly identical.^26^

In addition to these data, we obtained data of 9,893 JAGES participants with results of annual health checkups from five municipalities in Aichi Prefecture that participated in JAGES. After excluding participants with data missing for HbA1c, fasting blood glucose, casual blood glucose, or information of medication (*N* = 306) or SES variables (income, education and longest occupation) (*N* = 2,774), a total 6,813 (3,475 men and 3,338 women) participants were eligible for the analysis. We applied multiple imputation methods for the individuals having one or more missing data. Thus, the final study sample was 9,893 (4,471 men and 5,422 women). Approvals were received by the Ethics Committee in Research of Human Subjects at Nihon Fukushi University for the JAGES protocol (No. 10-05) and by the Ethics Committee of Chiba University, Faculty of Medicine for the use of the data (No. 1777).

### Measurement of diabetes and other metabolic risk factors

Annual health checkups are organized by local municipalities of Japan and performed at community centers or registered clinics or hospitals. Participant blood samples taken at annual checkups were analyzed following the standardized procedure of the Japan Society of Clinical Chemistry for HbA1c, fasting glucose, casual glucose, triglycerides (TG), and high-density lipoprotein (HDL) cholesterol. The HbA1c ratios were reported as the Japan Diabetes Society (JDS) values, then calculated for the values of the National Glycohemoglobin Standardization Program (NGSP) following a conversion formula.^27^ Blood pressure was measured twice in the right upper arm with participants in the sitting position, and the mean of the two measurements was recorded.

### Definition of diabetes and other metabolic risk factors

Based on the report from the Committee of the JDS on the Diagnostic Criteria of Diabetes Mellitus,^28^ we defined diabetes mellitus as having HbA1c of over 6.5% based on the NGSP and fasting blood sugar ≥126 mg/dL (≥7.0 mmol/L) and/or casual blood sugar ≥200 mg/dL (≥11.1 mmol/L). People regularly taking hypoglycemic agents or insulin were also considered to have diabetes. We used the following criteria of the Japanese Society of Internal Medicine^29^ to define other metabolic risks: hypertriglyceridemia (TG ≥150 mg/dL), low HDL cholesterol (HDL <40 mg/dL), or taking appropriate medication and hypertension (systolic blood pressure ≥140 mm Hg and/or diastolic blood pressure ≥90 mm Hg or taking antihypertensive drugs).

### Socioeconomic status

Information of participants’ annual household income, educational attainment, and longest occupation were collected using JAGES questionnaires. To adjust for differences in household size, we equivalized household income to per person in a household, dividing annual household income by the square root of the number of individuals per household. Because the income level of the participants was slightly higher than that of the entire JAGES population in 2010 (eTable 1), to apply the income level of the entire JAGES population, we categorized the study participants using quartiles of equivalized household income of all JAGES 2010 participants. We categorized the individuals into four groups: Q1 (low), JPY 1.25 million per year and below; Q2 (lower middle), JPY 1.251–1.944 million per year; Q3 (upper middle), JPY 1.945–3.061 million per year and Q4 (high), JPY 3.062 million per year and above. Educational status was categorized by the number of years of schooling (9 years or fewer, 10–12 years, and 13 years or more). Longest occupation was queried as follows: “What was the job that you did for most of your working life?” Responses included the following eight options: professional/technical, managerial, clerical, sales/service, skilled/manual, agriculture/forestry/fishery workers, other, and unemployed.^30^

### Covariates

We used categorical 5-year age groups, marital status, body mass index (BMI), other metabolic risks defined above, current smoking, current alcohol intake, physical activities, and dietary intake habits as covariates; these factors could be mediators of the association between SES and diabetes. Marital status was categorized as married, widowed, separated/unmarried, and other. BMI was classified into four groups: BMI <18.5, 18.5–24.9, 25.0–29.9, and ≥30.0. Other lifestyle factors included smoking status (nonsmoker or current smoker/ex-smoker), alcohol intake (nondrinker or drinker/ex-drinker), and walking time per day as physical activity (<30 min or ≥30 min). As for dietary intake habits, we included frequencies of the consumption of meat or fish (<1 servings/day or ≥1 servings/day) and fruit or vegetables (<1 servings/day or ≥1 servings/day).

### Statistical analysis

First, we calculated the prevalence of diabetes by the levels of socioeconomic indicators. Chi-squared test for sex was performed both in Table 1 and Table 2. Second, we performed multivariate Poisson regression analysis to calculate prevalence ratios (PRs) of diabetes and their 95% confidence intervals (CIs) across SES groups. To account for the potential biases due to the missing values, we used the multiple imputation techniques. All variables included in the analysis, such as the outcome variable, diabetes, explanatory variables, and covariates were imputed. Under a missing-at-random assumption, we created 10 imputed data using chained equation method, made analyses for each dataset, and combined the 10 results, using Rubin’s combination method.^31^^,^^32^ Under the chained equation method, we performed multinomial logistic regression for the categorical variables and ordinal logistic regression for the ordinal variables. We treated occupation and marital status as nominal variables and categorized diabetes, income, education, BMI, hypertension, triglyceridemia, smoking habit, alcohol intake, walking duration per day, and eating habit as ordinal variables, including dichotomous variables. Model 1 was adjusted for age and each SES indicators (income quartile, years of education, and longest occupation) separately. Model 2 was adjusted for age and all SES indicators. Model 3 was additionally adjusted for marital status, BMI, hypertension, low HDL, high TG, smoking status, alcohol intake, walking time per day, meat/fish intake, and fruit/vegetable intake.

**Table 1.** Participant characteristics (*N* = 6,813)

		Men	Women	

		(*N* = 3,475)	(*N* = 3,338)	*P*-value^a^
Diabetes	No	2,948 (84.8%)	2,996 (89.8%)	<0.001
	Yes	527 (15.2%)	342 (10.2%)	
Age, years	65–69	1,297 (37.3%)	1,335 (40.0%)	0.13
	70–74	1,213 (34.9%)	1,093 (32.7%)	
	75–79	606 (17.4%)	572 (17.1%)	
	80 and above	359 (10.3%)	338 (10.1%)	
Income quartile^b^	Q1	534 (15.4%)	795 (23.8%)	<0.001
	Q2	1,091 (31.4%)	903 (27.1%)	
	Q3	1,054 (30.3%)	897 (26.9%)	
	Q4	796 (22.9%)	743 (22.3%)	
Years of education	9 or less	1,556 (44.8%)	1,715 (51.4%)	<0.001
	10–12	1,284 (36.9%)	1,259 (37.7%)	
	13 and over	635 (18.3%)	364 (10.9%)	
Longest occupation	Professional/technical	968 (27.9%)	351 (10.5%)	<0.001
	Managerial	345 (9.9%)	26 (0.8%)	
	Clerical	371 (10.7%)	812 (24.3%)	
	Sales/service	309 (8.9%)	646 (19.4%)	
	Skilled/manual	970 (27.9%)	413 (12.4%)	
	Agriculture/forestry/fishery worker	202 (5.8%)	217 (6.5%)	
	Other	303 (8.7%)	572 (17.1%)	
	Unemployed	7 (0.2%)	301 (9.0%)	
Marital status	Married	3,159 (90.9%)	2,329 (69.8%)	<0.001
	Widowed	227 (6.5%)	861 (25.8%)	
	Separated/unmarried	65 (1.9%)	123 (3.7%)	
	Other/missing	24 (0.7%)	25 (0.7%)	
BMI, kg/m^2^	<18.5	77 (2.2%)	160 (4.8%)	<0.001
	18.5–24.9	1,671 (48.1%)	1,539 (46.1%)	
	25.0–29.9	573 (16.5%)	449 (13.5%)	
	≥30.0	36 (1.0%)	67 (2.0%)	
	Missing	1,118 (32.2%)	1,123 (33.6%)	
Hypertension	No	1,500 (43.2%)	1,406 (42.1%)	0.040
	Yes	1,659 (47.7%)	1,675 (50.2%)	
	Missing	316 (9.1%)	257 (7.7%)	
High TG	No	2,208 (63.5%)	2,145 (64.3%)	0.54
	Yes	1,267 (36.5%)	1,193 (35.7%)	
Low HDL	No	2,682 (77.2%)	2,673 (80.1%)	0.004
	Yes	793 (22.8%)	665 (19.9%)	
Smoking status	No	824 (23.7%)	2,851 (85.4%)	<0.001
	Smoker/ex-smoker	2,433 (70.0%)	192 (5.8%)	
	Missing	218 (6.3%)	295 (8.8%)	
Alcohol intake	Drinker/ex-drinker	2,248 (64.7%)	631 (18.9%)	<0.001
	None	1,036 (29.8%)	2,543 (76.2%)	
	Missing	191 (5.5%)	164 (4.9%)	
Walking time, min/day	<30	889 (25.6%)	989 (29.6%)	<0.001
	≥30.0	2,447 (70.4%)	2,183 (65.4%)	
	Missing	139 (4.0%)	166 (5.0%)	
Meat/fish intake, servings/day	≥1	1,138 (32.7%)	1,376 (41.2%)	<0.001
	<1	2,133 (61.4%)	1,796 (53.8%)	
	Missing	204 (5.9%)	166 (5.0%)	
Fruit/vegetable intake, servings/day	≥1	2,478 (71.3%)	2,773 (83.1%)	<0.001
	<1	812 (23.4%)	406 (12.2%)	
	Missing	185 (5.3%)	159 (4.8%)	

BMI, body mass index; HDL, high-density lipoprotein; TG, triglyceride.

^a^Chi-squared test for sex.

^b^Income quartile calculated by all participants in JAGES2010 (‘Low’ −1.250, ‘Middle-low’ 1.251–1.944, ‘Middle-high’ 1.945–3.061, ‘High’ 3.062− million yen per year).

**Table 2.** Prevalence of diabetes mellitus by socioeconomic status and sex (*N* = 6,813)

	*N*	Men	*N*	Women
**Income quartile^a^**				
Q1	534	94 (17.6%)	795	93 (11.7%)
Q2	1,091	147 (13.5%)	903	105 (11.6%)
Q3	1,054	166 (15.7%)	897	86 (9.6%)
Q4	796	120 (15.1%)	743	58 (7.8%)
*P*-value		0.16		0.03
**Years of formal education**				
9 or less	1,556	237 (15.2%)	1,715	187 (10.9%)
10–12	1,284	196 (15.3%)	1,259	113 (9.0%)
13 and over	635	94 (14.8%)	364	42 (11.5%)
*P*-value		0.96		0.16
**Longest occupation**				
Professional/technical	968	145 (15.0%)	351	43 (12.3%)
Managerial	345	56 (16.2%)	26	5 (19.2%)
Clerical	371	59 (15.9%)	812	71 (8.7%)
Sales/service	309	51 (16.5%)	646	55 (8.5%)
Skilled/manual	970	132 (13.6%)	413	47 (11.4%)
Agriculture/forestry/fishery workers	202	28 (13.9%)	217	21 (9.7%)
Other	303	53 (17.5%)	572	62 (10.8%)
Unemployed	7	3 (42.9%)	301	38 (12.6%)

*P*-values were calculated using Chi-squared test.

^a^Income quartile calculated by all participants in JAGES2010 (‘Low’ –1.250, ‘Middle-low’ 1.251–1.944, ‘Middle-high’ 1.945–3.061, ‘High’ 3.062– million yen per year).

Preliminary analysis showed that the interaction terms for sex and socioeconomic indicators were not statistically significant (*P*-value for the interaction term between income and sex = 0.18, *P*-value for the interaction term between education and sex = 0.20). However, as Hawkes et al mentioned,^33^ irrespective of the statistical significance, the gender-stratified analysis is essential to address the determinants of ill health by gender. Accordingly, we decided to analyze the data stratified by sex. Also, to investigate the validity of our missing-at-random assumption for multiple imputations, we conducted a sensitivity analysis using the complete case dataset (eTable 2). We used Stata/SE version 13.1 (StataCorp LLC, College Station, TX, USA) for the analyses.

## RESULTS

Among our study participants, 15.2% of men and 10.2% of women had diabetes. Around 70% of both men and women were under 75 years old (Table 1). A total 15.4% of men and 23.8% of women were in the low-income quartile; these percentages were 22.9% and 22.3%, respectively, for the high-income quartile. With regard to years of formal education, 44.8% of men and 51.4% of women had nine years or fewer years of schooling, and 18.3% of men and 10.9% of women had 13 years or more. Distributions of longest occupation were entirely different between men and women. Compared with the entire JAGES 2010 population, our study participants were older; had slightly higher income and lower education levels; and there were more married and physically active participants, as well as more alcohol drinkers (eTable 1).

The prevalence of diabetes by income quartile among men was 17.6% in Q1 (lowest income), 13.5% in Q2, 15.7% in Q3, and 15.1% in Q4 (highest income) (*P* = 0.16); among women, prevalence values were 11.7% in Q1, 11.6% in Q2, 9.6% in Q3, and 7.8% in Q4 (*P* = 0.03) (Table 2). Education- and occupation-related gradients were not observed in the population.

The results of multivariate analysis showed that among women, an income-based gradient was observed in the prevalence of diabetes. Compared with Q4 (highest income category), PRs of diabetes for Q1, Q2, and Q3 were 1.43 (95% CI, 1.07–1.90), 1.33 (95% CI, 1.01–1.75), and 1.22 (95% CI, 0.91–1.64) (*P* for trend = 0.01; Table 3, women, model 1). After mutually adjusting for each SES factor, the PRs of Q1, Q2, and Q3 compared to Q4 were 1.42 (95% CI, 1.06–1.90), 1.33 (95% CI, 1.00–1.76), and 1.23 (95% CI, 0.91–1.65), respectively (*P* for trend = 0.016; Table 3, women, model 2). Even after adjustment for marital status, BMI, other metabolic risk factors, and lifestyle factors, the association was not attenuated (Q1: PR 1.43; 95% CI, 1.07–1.92, Q2: PR 1.32; 95% CI, 0.99–1.76; Q3: PR 1.22; 95% CI, 0.91–1.65; *P* for trend = 0.01; Table 3, women, model 3). No socioeconomic gradient was observed among men (Table 3).

**Table 3.** Prevalence ratios and 95% confidence intervals for diabetes mellitus by sex with multiple imputation (*N* = 9,893)

	Men (*N* = 4,471)			Women (*N* = 5,422)		
	
	Model 1	Model 2	Model 3	Model 1	Model 2	Model 3
**Income quartile**						
Q1 (lowest)	1.16 (0.90–1.50)	1.16 (0.88–1.52)	1.18 (0.89–1.56)	1.43 (1.07–1.90)	**1.42 (1.06–1.90)**	**1.43 (1.07–1.92)**
Q2	0.93 (0.74–1.17)	0.94 (0.74–1.19)	0.96 (0.76–1.22)	1.33 (1.01–1.75)	**1.33 (1.00–1.76)**	**1.32 (0.99–1.76)**
Q3	1.02 (0.79–1.30)	1.03 (0.80–1.32)	1.02 (0.80–1.31)	1.22 (0.91–1.64)	1.23 (0.91–1.65)	1.22 (0.91–1.65)
Q4 (Highest)	1 (referent)	1 (referent)	1 (referent)	1 (referent)	1 (referent)	1 (referent)
Trend *P*	0.52	0.55	0.44	**0.01**	**0.016**	**0.01**
**Years of formal education**						
9 years or less	0.97 (0.78–1.20)	0.99 (0.78–1.24)	1.01 (0.80–1.27)	1.03 (0.77–1.39)	0.97 (0.72–1.33)	0.94 (0.69–1.28)
10–12	1.00 (0.81–1.25)	1.02 (0.81–1.27)	1.02 (0.81–1.27)	0.96 (0.71–1.30)	0.96 (0.71–1.31)	0.95 (0.69–1.29)
13+	1 (referent)	1 (referent)	1 (referent)	1 (referent)	1 (referent)	1 (referent)
Trend *P*	0.701	0.84	0.99	0.56	0.96	0.72
**Longest occupation**						
Professional/Technical	1 (referent)	1 (referent)	1 (referent)	1 (referent)	1 (referent)	1 (referent)
Managerial	0.98 (0.73–1.32)	0.98 (0.73–1.33)	0.98 (0.73–1.33)	1.20 (0.60–2.41)	1.21 (0.60–2.41)	1.20 (0.60–2.40)
Clerical	1.03 (0.77–1.38)	1.03 (0.77–1.38)	1.05 (0.78–1.42)	0.84 (0.59–1.20)	0.86 (0.60–1.24)	0.88 (0.62–1.26)
Sales/Service	1.05 (0.79–1.41)	1.04 (0.78–1.39)	1.01 (0.75–1.35)	0.79 (0.55–1.12)	0.77 (0.53–1.10)	0.75 (0.53–1.08)
Skilled/Manual	0.86 (0.69–1.08)	0.87 (0.69–1.09)	0.86 (0.68–1.08)	0.99 (0.69–1.43)	0.97 (0.66–1.41)	0.95 (0.65–1.39)
Agriculture/Forestry/Fishery workers	0.91 (0.63–1.31)	0.89 (0.62–1.30)	0.88 (0.61–1.29)	0.86 (0.55–1.33)	0.83 (0.53–1.29)	0.81 (0.52–1.27)
Others	1.12 (0.84–1.48)	1.10 (0.82–1.47)	1.08 (0.81–1.45)	1.03 (0.72–1.46)	0.99 (0.69–1.42)	0.98 (0.68–1.41)
Unemployed	1.64 (0.68–3.94)	1.60 (0.66–3.90)	1.59 (0.66–3.86)	1.03 (0.68–1.57)	1.00 (0.65–1.54)	0.98 (0.64–1.50)

Model 1 was adjusted for adjusted for income quartile, years of formal education and longest occupation separately with age.

Model 2 was adjusted for income quartile, years of formal education, longest occupation, and age.

Model 3 was additionally adjusted for marital status, BMI, hypertension, low HDL, high TG, smoking status, alcohol drinking habit, walking time per day, and meat/fish intake and vegetable intake.

The estimates based on our sensitivity analysis using complete case data were mostly identical to our original analysis with slightly smaller PRs and wider CIs (eTable 2).

## DISCUSSION

Using the large-scale data of Japanese older adults, we found two major findings on the social inequality in objectively measured diabetes: 1) the clear income gradient in diabetes prevalence was only observed among women but not among men; and 2) among men and women, there was no clear gradient in diabetes prevalence by years of education and longest occupation.

The socioeconomic gradient was potentially more marked among women, which was consistent with recent studies in other countries.^12^^,^^21^ Robbins et al have proposed, as potential reasons, that women culturally have difficulties in health care access than men, fewer opportunities for regular exercise, unhealthy lifestyle behaviors, disadvantaged nutritional factors, more psychological stress, more depression, and more negative pre- or peri-natal environmental factors.^18^ Other scholars have suggested the different roles of obesity in the association between income and diabetes by sex. In a Swedish study, Agardh et al found that among the low-income group, BMI explained their excess risk for subjectively diagnosed type 2 diabetes by 21% among men and 35% among women.^3^ Nonetheless, a study from Canada that investigated the association between self-reported diabetes and SES found that BMI did not explain the associations between income and diabetes both among men and women.^20^ In the present study, further adjustment for covariates, including BMI, did not substantially alter the association between income and diabetes for both sexes. To clarify the reasons for the sex difference, further studies are needed.

We found a gradient in diabetes by income but not by education or occupation. In theory, income has both materialistic and psychosocial functions, and they may explain the income gradient in diabetes distinctively. First, low income means limited access to material goods and services to prevent diabetes, such as balanced diet and necessary preventive care.^34^ Second, the access limitation also leads to the social isolation and exclusion because of the lack of opportunities for social interactions, leading to mental stresses. Stress science and endocrinological studies have suggested the direct effects of stress hormones on blood glucose levels and insulin intolerance, as well as health behaviors.^34^ Potential gender differences in our result could be explained by the psychosocial functions of income, including health beliefs, attitudes, and lifestyles, which may differ between men and women even at the same income levels.^21^ Specifically, as suggested by Saito et al, the loss of social interactions due to the lack of income might affect women more than men among Japanese older adults.^35^ Lastly, although the detailed mechanisms are unknown, sex differences in the gene-related tolerance for diabetes may also explain the stronger association among women found in our study.^36^^,^^37^

Although we found a gradient in diabetes by income but not by education or occupation, these results were inconsistent with those among young or middle-aged adults^18^ but consistent with results from older populations.^6^ Socioeconomic status in older people should be interpreted differently from that at younger ages.^38^ In many countries, older people are likely to have lower educational attainment. Among our study participants, the percentage of people with university or higher level educations was small in the age group investigated: 18.3% for men and 10.9% for women (Table 1). However, the university entrance rate in Japan was 56.6% among men and 57.1% among women in 2016.^39^ Consequently, the number of older people with high educational attainment is small, resulting in less statistical power to capture the association between education level and diabetes. The null finding between longest occupation and diabetes among men and women may be explained by weak statistical power owing to small sample sizes of each occupational category. For example, among men, the PR of diabetes among unemployed compared with professional/technical workers was large (PR 1.64; 95% CI, 0.68–3.94), which is in line with known occupation-based health disparities around the world (Table 3).^40^ Alternatively, the survivor effect could alter the association between education, previous occupation, and diabetes, given that those who are socioeconomically deprived are less likely to survive; this tendency could be stronger in Japan, where many people experienced the life-threatening post-war period.^41^

Apart from those discussed above, four additional limitations in our study should be mentioned. First and foremost, owing to the cross-sectional nature of the study, we cannot exclude the possibility of reverse causation (ie, diabetes causes reduced income). Second, generalizability is limited, as our data were obtained only from regions of central Japan and the study does not include older people with long-term care insurance. Third, selection bias should also be discussed. Our study participants were more health conscious than the general population, as participants were limited to those who underwent health checkups. In Japan, about 38% of the population received health checkups in 2010.^42^ Underestimation of the magnitude of SES-related health associations found in this study may be owing to this bias. Nonetheless, our sensitivity analysis using complete cases only showed the same income-based gradient, suggesting the missing did not induce a critical bias to the levels of the income-based gradient in diabetes. Finally, we did not evaluate the health gradient stemming from other SES indicators, including previously suggested indicators associated with health: wealth,^5^ relative deprivation,^43^ and social exclusion.^35^ Specifically, future studies should evaluate the wealth-based gradient given that older adults are more likely to rely on savings or other similar financial resources rather than regular income, which mostly consists of a government pension.

In conclusion, we found a clear income-based gradient in diabetes among Japanese older adults and the gradient was potentially more remarkable among women, but this was not the case for education and longest occupation. This was the first large-scale study clarifying the socioeconomic disparity in diabetes among Japanese older population. Given the findings of this study, monitoring income gradient in diabetes is important in public health actions, even in older populations. Future longitudinal and intervention studies should evaluate the causal link of income to diabetes onset, determine the mechanisms of the potential sex differences in the income/diabetes association, and identify ways to mitigate the income-based inequality.

## APPENDIX A. SUPPLEMENTARY DATA

eTable 1. Comparison of characteristics between the study sample and overall JAGES 2010 population

eTable 2. Prevalence ratios (95% confidence intervals) for diabetes mellitus by sex with complete cases for SES^*^ (N = 6,813)
