# Supplementary material for: Socioeconomic Disparity in the Prevalence of Objectively Evaluated Diabetes Among Older Japanese Adults: JAGES Cross-Sectional Data in 2010
Source: J Epidemiol. 2019 Aug 5;29(8):295–301. doi: 10.2188/jea.JE20170206 (PMC6614078; doi:10.2188/jea.JE20170206)
Supplement: Supplementary file 1 [file je-29-295-s001.pdf]

eTable 1. Comparison of characteristics between the study sample and overall JAGES 2010 population

		Men		Women	
		Study sample	JAGES 2010	Study sample	JAGES 2010
		N=3,475	N=47,269	N=3,338	N=55,545
		n (%)	n (%)	n (%)	n (%)
**Age, years**	65–69	1297 (37.3%)	13691 (29.0%)	1335 (40.0%)	14984 (27.0%)
	70–74	1213 (34.9%)	13776 (29.1%)	1093 (32.7%)	15940 (28.7%)
	75–79	606 (17.4%)	10727 (22.7%)	572 (17.1%)	12657 (22.8%)
	80 and above	359 (10.3%)	9075 (19.2%)	338 (10.1%)	11964 (21.5%)
**Income quartile**	Q1 (low)	534 (15.4%)	8945 (18.9%)	795 (23.8%)	13395 (24.1%)
	Q2 (lower middle)	1091 (31.4%)	10906 (23.1%)	903 (27.1%)	9262 (16.7%)
	Q3 (upper middle)	1054 (30.3%)	10404 (22.0%)	897 (26.9%)	9542 (17.2%)
	Q4 (high)	796 (22.9%)	10766 (22.8%)	743 (22.3%)	9793 (17.6%)
	Missing		6248 (13.2%)		13553 (24.4%)
**Educational level**	9 years or less	1556 (44.8%)	20679 (43.7%)	1715 (51.4%)	27519 (49.5%)
	10–12 years	1284 (36.9%)	15088 (31.9%)	1259 (37.7%)	19086 (34.4%)
	13 years and over	635 (18.3%)	10468 (22.1%)	364 (10.9%)	7000 (12.6%)
	Missing		1034 (2.2%)		1940 (3.5%)
**Occupation**	Professional/technical	968 (27.9%)	9919 (21.0%)	351 (10.5%)	4477 (8.1%)
	Managerial	345 (9.9%)	5048 (10.7%)	26 (0.8%)	483 (0.9%)
	Clerical	371 (10.7%)	4255 (9.0%)	812 (24.3%)	9100 (16.4%)
	Sales/service	309 (8.9%)	5387 (11.4%)	646 (19.4%)	8287 (14.9%)
	Skilled/manual	970 (27.9%)	8797 (18.6%)	413 (12.4%)	4049 (7.3%)
	Agriculture/forestry/fishery worker	202 (5.8%)	4032 (8.5%)	217 (6.5%)	4101 (7.4%)
	Other	303 (8.7%)	4611 (9.8%)	572 (17.1%)	7924 (14.3%)
	Unemployed	7 (0.2%)	355 (0.8%)	301 (9.0%)	5298 (9.5%)
	Missing		4865 (10.3%)		11826 (21.3%)

**Marital status**	Married	3159 (90.9%)	40285 (85.2%)	2329 (69.8%)	31772 (57.2%)
	Widowed	227 (6.5%)	4031 (8.5%)	861 (25.8%)	18689 (33.6%)
	Separated/unmarried	65 (1.9%)	1925 (4.1%)	123 (3.7%)	3461 (6.2%)
	Others/missing	24 (0.7%)	1028 (2.2%)	25 (0.7%)	1623 (2.9%)
**Smoking status**	No	824 (23.7%)	11637 (24.6%)	2851 (85.4%)	43740 (78.7%)
	Smoker/ex-smoker	2433 (70.0%)	31874 (67.4%)	192 (5.8%)	4143 (7.5%)
	Missing	218 (6.3%)	3758 (8.0%)	295 (8.8%)	7662 (13.8%)
**Alcohol intake**	None	1036 (29.8%)	16205 (34.3%)	2543 (76.2%)	43433 (78.2%)
	Drinker/ex-drinker	2248 (64.7%)	28071 (59.4%)	631 (18.9%)	8388 (15.1%)
	Missing	191 (5.5%)	2993 (6.3%)	164 (4.9%)	3724 (6.7%)
**Walking time**	<30 min/day	889 (25.6%)	14271 (30.2%)	989 (29.6%)	17978 (32.4%)
	≥30 min/day	2447 (70.4%)	30303 (64.1%)	2183 (65.4%)	33172 (59.7%)
	Missing	139 (4.0%)	2695 (5.7%)	166 (5.0%)	4395 (7.9%)
**Meat/fish intake**	≥1 servings/day	1138 (32.7%)	16548 (35.0%)	1376 (41.2%)	22130 (39.8%)
	<1 servings/day	2133 (61.4%)	27521 (58.2%)	1796 (53.8%)	29843 (53.7%)
	Missing	204 (5.9%)	3200 (6.8%)	166 (5.0%)	3572 (6.4%)
**Fruit/vegetable intake**	≥1 servings/day	2478 (71.3%)	33149 (70.1%)	2773 (83.1%)	43750 (78.8%)
	<1 servings/day	812 (23.4%)	11209 (23.7%)	406 (12.2%)	8632 (15.5%)
	Missing	185 (5.3%)	2911 (6.2%)	159 (4.8%)	3163 (5.7%)

eTable 2. Prevalence ratios (95% confidence intervals) for diabetes mellitus by sex with complete cases for SES* (N=6,813)

	Men (N=3,475)			Women (N=3,338)		
	**Model 1**	**Model 2**	**Model 3**	**Model 1**	**Model 2**	**Model 3**
**Income quartile**						
Q1 (lowest)	1.15 (0.88-1.51)	1.14 (0.86-1.51)	1.14 (0.86-1.53)	**1.52 (1.09-2.11)**	**1.50 (1.07-2.10)**	**1.48 (1.05-2.09)**
Q2	0.89 (0.70-1.13)	0.90 (0.70-1.15)	0.92 (0.71-1.17)	**1.48 (1.07-2.04)**	**1.49 (1.08-2.06)**	**1.44 (1.04-2.00)**
Q3	1.04 (0.82-1.32)	1.05 (0.83-1.33)	1.02 (0.80-1.29)	1.23 (0.88-1.71)	1.25 (0.89-1.74)	1.25 (0.89-1.75)
Q4 (highest)	1 (referent)	1 (referent)	1 (referent)	1 (referent)	1 (referent)	1 (referent)
p trend	0.80	0.86	0.73	0.006	**0.011**	**0.02**
**Years of formal education**						
9 or less	1.01 (0.80-1.29)	1.07 (0.82-1.39)	1.10 (0.84-1.43)	0.96 (0.69-1.34)	0.92 (0.65-1.30)	0.82 (0.57-1.17)
10–12	1.04 (0.81-1.33)	1.07 (0.83-1.37)	1.04 (0.81-1.34)	0.78 (0.55-1.11)	0.80 (0.55-1.14)	0.75 (0.52-1.07)
13 and over	1 (referent)	1 (referent)	1 (referent)	1 (referent)	1 (referent)	1 (referent)
p trend	0.97	0.64	0.46	0.53	0.92	0.60
**Longest occupation**						
Professional/technical	1 (referent)	1 (referent)	1 (referent)	1 (referent)	1 (referent)	1 (referent)
Managerial	1.08 (0.79-1.47)	1.09 (0.80-1.50)	1.13 (0.82-1.56)	1.65 (0.65-4.18)	1.64 (0.65-4.14)	1.60 (0.63-4.08)
Clerical	1.06 (0.78-1.43)	1.06 (0.78-1.44)	1.12 (0.83-1.53)	0.72 (0.50-1.06)	0.78 (0.53-1.14)	0.84 (0.57-1.24)
Sales/service	1.10 (0.80-1.51)	1.08 (0.79-1.49)	1.07 (0.78-1.48)	0.70 (0.47-1.04)	0.68 (0.45-1.02)	0.69 (0.46-1.04)
Skilled/manual	0.90 (0.71-1.15)	0.90 (0.70-1.14)	0.87 (0.68-1.11)	0.94 (0.62-1.42)	0.90 (0.59-1.37)	0.95 (0.62-1.45)
Agriculture/forestry/fishery worker	0.90 (0.60-1.35)	0.86 (0.57-1.30)	0.86 (0.57-1.31)	0.82 (0.49-1.39)	0.79 (0.46-1.36)	0.82 (0.48-1.40)
Other	1.16 (0.85-1.59)	1.13 (0.82-1.56)	1.09 (0.79-1.51)	0.91 (0.61-1.34)	0.86 (0.57-1.28)	0.91 (0.61-1.37)
Unemployed	2.69 (0.85-8.47)	2.58 (0.82-8.18)	2.88 (0.89-9.35)	1.07 (0.69-1.66)	1.06 (0.68-1.66)	1.06 (0.68-1.66)

*SES, socioeconomic status.

Model 1 was adjusted for income quartile, years of formal education and longest occupation separately with age.

Model 2 was mutually adjusted for income quartile, years of formal education, longest occupation, and age.

Model 3 was additionally adjusted for marital status, BMI, hypertension, low HDL, high TG, smoking status, alcohol intake, walking time per day, meat/fish intake and fruit/vegetable intake.
